# Obstetrics and Gynecology

**Published:** 1893-07

**Authors:** 


### OBSTETRICS AND GYNECOLOGY.

EDITED BY DR. VIRGIL O. HARDON.

### ACCUMULATION OF PUS IN THE FALLOPIAN TUBES.

After discussing the pathology of this condition Dr. G. Wackerhagen says:

With regard to management, other than laparotomy, it has been suggested that a sound passed from the uterus into the Fallopian tube would much facilitate treatment, but I think that this is generally considered a very questionable procedure for fear of puncturing the tube or forcing its contents through the opening of the fimbriated extremity.

By some surgeons a method of pressing the contents of the tumor into the uterus has been practiced, but this is also objectionable, for the reason that the pus might be forced into the peritoneal cavity instead of into the uterus. Either of these plans of treatment might endanger the life of the patient from shock or cause a fatal peritonitis.

Then again, puncturing the abscess through the vagina or abdominal wall has been advocated, and here, also, we find objections.

1st. The difficulty of maintaining effectual drainage through the vagina, and

2d. The danger of wounding the intestines when puncturing through the abdominal wall.

In cases where the patient has been under constant and early observation, where there has been little time for extensive adhesions, I most earnestly advocate the following treatment:

The vulva, vagina, and surrounding parts having been cleansed as thoroughly as possible by brush, soap and water, are then irrigated with bichloride solution 1-2000, and a rubber or steel applicator wound with cotton and saturated with pure carbolic acid is passed into the uterus. A small uterine dilator, which has also been dipped in pure carbolic acid, is then introduced into the cervical canal, and gradual dilatation is commenced. This is continued daily until the uterine catheter can be easily introduced, and irrigation practiced after each dilatation. As soon as the catheter can be easily passed, pus begins to flow, and the sac is gradually reduced in size until it disappears. After each application a pledget of absorbent cotton, saturated with boro-glycerate of alum, is placed against the cervix and allowed to remain until the next morning, when it is removed, and the vagina is irrigated with boro-salicylic solution; this irrigation is continued every eight hours.

I am unable to explain the reason, but it has been my experience that as soon as thorough dilatation has been accomplished, pus begins to flow copiously and the distended tube gradually to diminish, until all pathological condition disappears. It may be possible that the dilatation of the muscular fibres at the cervix causes a simultaneous relaxation at the tubal attachment of the uterus.—*Brooklyn Medical Journal.*

---

### SUBCUTANEOUS CHLORAL INJECTIONS IN PUERPERAL ECLAMPSIA.

Deshages, of Orleans, considers the subcutaneous injection of chloral a valuable method of its administration in puerpera eclampsia, where it is impossible to administer it by the mouth and there is a rectal intolerance. From his experience in seven different cases, Deshages believes it to be the safest working method of administering chloral. In all cases an initial dose of from 7½ to 15 grains sufficed at once to quiet the patient and to lengthen the period between the seizures. The total quantity used was usually from 45 grains to 1 drachm, given either in three doses at intervals of eight to ten hours, or following a large initial dose, hourly, 1½ to 3 grains. The largest quantity used was 75 grains. The effect of the large initial dose differs in individuals, according to the depth of the uremic intoxication, the condition of the os, the pains, to the nearness of the delivery. The injections are only to be used in comatose conditions, on account of the severe pains accompanying them, unless morphia or cocaine are added. The strength of the solution should not be greater than 1.10, and the syringeful should be very slowly injected.

Deshages has also used this method in the eclampsias of non-puerperal origin with good results.—*University Medical Magazine.*

---

### SYMPHYSEOTOMY COMPARED WITH ITS SUBSTITUTES, WITH REFERENCE TO A CASE.

The author said little over a century ago the Italian surgeon, Sigault, introduced a symphyseotomy. Of the first forty-four cases following the introductory operation fifteen women and thirty-two children were lost, while an ununited symphysis, an injured sacro-iliac synchondrosis or a vesico-vaginal fistula were not unknown complications. He then reported a case on which he had operated successfully. The experience of the last few years had demonstrated that there should be no objection to symphyseotomy in suitable cases and when properly performed. The pelvic diameters are increased sufficiently to allow the child to pass. The sacro-iliac synchondroses are not interfered with, while the pubic bones reunite with marked rapidity and firmness. The operation is particularly indicated in cases where there are no ankylosed pelvic joints, where there are no obstructive growths and where the antero-posterior pelvic diameters range between two and threefourths and three and three-fourths inches. It is contraindicated in either a Robert's or Nægele's pelvis, where the conjugate diameter is less than two and three-fourths inches and where there is a dead fœtus. The operation should not be preceded by frequent unsuccessful attempts to deliver with forceps, and it should be done early. Whether the patient be in a tenement house or a well equipped hospital, as soon as the os is sufficiently dilated the operation should be done. Death follows delays and exposures necessarily caused while removing the patient from her home to the hospital.—*Dr. H. McKennan in Journal of American Medical Association.*

---

### THE RELATION OF THE MENOPAUSE TO CERTAIN COINCIDENT DISORDERS IN WOMEN.

Bigg (*Merck's Bulletin,* January, 1893); conclusions:

1. In women whose nutrition has uniformly approximated the normal standard, and who come to this period unhampered by pre-existing ailments, the final cessation of menstruation occurs without material disturbance of the functional harmony, and is often of cosmetic advantage.

2. The association of morbid conditions with the menopause is *accidental*—and the result, usually, of *antecedent* causes, especially of unphysiological living.

3. The influence of *perfect nutrition and natural living* during the pre-menstrual and adolescent years, upon the after-life of woman, is of the most salutary and far-reaching kind. Hence:

4. It is of the utmost importance that medical men should unceasingly impress this fact upon the mothers of girls, and initiate *a natural development* of their physical organism by the use of wholesome and nutritious diet at regular intervals, by abundance of outdoor exercise, by the avoidance of late hours, and by a system of school-instruction which shall be graduated to a rational output of mental energy by the adolescent faculties.

